# Family Meetings in the Intensive Care Unit During the Coronavirus Disease 2019 Pandemic

**DOI:** 10.1177/1049909120973431

**Published:** 2020-11-19

**Authors:** Gina M. Piscitello, Corinna M. Fukushima, Anna K. Saulitis, Katherine T. Tian, Jennifer Hwang, Shreya Gupta, Mark Sheldon

**Affiliations:** 1 Section of Palliative Medicine, 12245 Rush Medical College, Chicago, IL, USA; 2 12245 Rush Medical College, Chicago, IL, USA; 3 Rush University Medical Center, Chicago, IL USA; 4 Department of Medicine, 12245 Rush Medical College, Chicago, IL, USA; 5 Department of Philosophy, 3270 Northwestern University, Evanston, IL, USA

**Keywords:** family meetings, intensive care unit, telehealth, critical care, patient-physician communication, COVID-19

## Abstract

**Purpose:**

Visitor restrictions during the COVID-19 pandemic limit in-person family meetings for hospitalized patients. We aimed to evaluate the quantity of family meetings by telephone, video and in-person during the COVID-19 pandemic by manual chart review. Secondary outcomes included rate of change in patient goals of care between video and in-person meetings, the timing of family meetings, and variability in meetings by race and ethnicity.

**Methods:**

A retrospective cohort study evaluated patients admitted to the intensive care unit at an urban academic hospital between March and June 2020. Patients lacking decision-making capacity and receiving a referral for a video meeting were included in this study.

**Results:**

Most patients meeting inclusion criteria (N = 61/481, 13%) had COVID-19 pneumonia (n = 57/61, 93%). A total of 650 documented family meetings occurred. Few occurred in-person (n = 70/650, 11%) or discussed goals of care (n = 233/650, 36%). For meetings discussing goals of care, changes in patient goals of care occurred more often for in-person meetings rather than by video (36% vs. 11%, p = 0.0006). The average time to the first goals of care family meeting was 11.4 days from admission. More documented telephone meetings per admission were observed for White (10.5, SD 9.5) and Black/African-American (7.1, SD 6.6) patients compared to Hispanic or Latino patients (4.9, SD 4.9) (p = 0.02).

**Conclusions:**

During this period of strict visitor restrictions, few family meetings occurred in-person. Statistically significant fewer changes in patient goals of care occurred following video meetings compared to in-person meetings, providing support limiting in-person meetings may affect patient care.

## Introduction

Strict visitor restrictions for patients requiring hospitalization during the Coronavirus Disease 2019 (COVID-19) pandemic is a unique situation that affects both patient and families.^1,2^ Medical decision-making is especially altered for patients lacking decision-making capacity who often cannot have family at bedside to advocate for them and require alternate decision-makers to make decisions on their behalf.^3^ Lack of in-person visitors causes concern that breakdown or mistakes in communication between clinicians and families may occur, and patient mortality may be affected if families are less engaged in patient care.^4,5^ Known benefits of family support interventions in the intensive care unit (ICU) such as improved quality of communication and perceived patient centered care also are at risk with visitor restrictions.^6^ Since the beginning of the pandemic, use of video family meetings by medical providers has increased.^7^ Although literature exists evaluating implementation and use of video meetings for patients and families, little is known about the use and frequency of video meetings to help with decision making for adult inpatients lacking decision-making capacity when compared to in-person meetings.^8-11^

The primary aim of this study was to evaluate the quantity of family meetings by telephone, video, and in-person for patients receiving care in the intensive care unit during strict visitor restriction policies due to the COVID-19 pandemic. Secondary aims of this study include evaluating for changes in patient care following family meetings by video or in-person, the timing of family meetings during patient admission, racial or ethnic differences in the use of meetings, and the timing and use of palliative medicine consultation for these patients.

## Methods

### Study Design and Population

A retrospective observational cohort study evaluating the use of telephone, video, and in-person family meetings was performed for critically ill patients during the COVID-19 pandemic. Patients admitted to the medical or cardiac ICU at an urban, academic medical center between March 27, 2020 and June 30, 2020 were eligible for inclusion in this study. Inclusion criteria were patients who were presumed to lack decision-making capacity for at least 1 day based on documented clinician notes that the patient was not alert or did not respond to questions when asked. We chose this criteria as we wanted to assess a patient population where family meetings most likely occurred during a period of strict visitor restrictions, as the aim of this study was to evaluate use of family meetings by telephone, video or in-person. Patients who lacked a mental status during their admission at some point could not consent to decision-making themselves and thus likely required family meetings to assess patient values and preferences to make medical decisions. Although it is possible patients without decision-making capacity have thorough advance directives with detailed documentation of their preferences potentially negating need for a decision-maker to help with medical decisions, we believe decision-makers still likely were contacted by the medical team to confirm the patient’s written values and preferences have not changed since they were documented and thus a family meeting was likely conducted. Many patients with decision-making capacity also benefit from family meetings to assist in identifying their values and preferences for medical care, but we chose for this study not to evaluate patients with decision-making capacity throughout their admission because legally these patients can consent to decisions without the aid of family, although this may not be optimal for their care. We were concerned including patients with decision-making capacity would significantly alter our results as we presumed these patients to be less likely to have family meetings due to strict visitor restrictions and decreased time of medical staff to talk with families due to significant rises in the quantity and severity of patients they were required to care for. Inclusion criteria also included patient, family or medical staff request for video family meetings. We chose to restrict the study population to patients with access to video visits as one aim of our study was to assess for differences in the use of video family meetings by video versus in-person. Staff were educated to contact case management for assistance with organizing video visits. Thus, requests for video visits were assessed by documented referrals to case management to organize video meetings recorded by case management throughout the study period and by documentation in the electronic medical record.

### Chart Review and Determination of a Family Meeting

Manual chart review of all notes in each patient’s medical record were assessed to evaluate for any documented discussion between medical staff, patients, and families by telephone, video, or in-person. Family meetings were broadly defined as any meeting between medical staff and a patient or family including brief updates. Goals of care family meetings were more strictly defined as any meeting between medical staff and the patient or family including discussion of the patient’s values or preferences for medical decisions. Families were defined as any alternate decision-maker, relative, or friend of the patient. Medical staff included physicians, advanced practice providers, nurses, chaplains, social workers, case managers, and medical students. Documented attempts at family meetings were counted as actual family meetings, as the meeting would have occurred if the family was reached. All ICU teams were told they were required to provide daily updates to patient families for all patients in this study. Change in goals of care was coded to include a change in code status or a decision to stop or start life support measures. Manual chart review was completed by 5 reviewers (G.M.P., C.M.F., A.K.S., K.T.T., and J.H.). Manual chart review included looking at every clinician note in the medical record during the entire admission of each patient to assess for the variables listed below.

### Variables and Data Source

Data collected from the chart included all documented family meetings including the time, mode, participants, and whether goals of care was discussed. Patient data collected included age, sex, race/ethnicity, primary diagnosis, discharge location, date of admission and discharge, insurance status, documentation of mental status, date of intubation and tracheostomy placement. Other data collected included date of palliative medicine consult and inclusion of palliative medicine in family meetings to evaluate the secondary aim of whether the use of palliative medicine impacts the frequency and outcome of family meetings. These variables were chosen to fully assess the primary and secondary outcomes of this study. We chose to collect additional patient characteristics to better describe the study population assessing factors such as the patient’s diagnosis and insurance status as an indicator for socioeconomic status.

### Data Analysis

We described continuous variables using mean and standard deviation. One-way analysis of variance (ANOVA) was used to compare the difference among race and ethnicity in the performance of documented family meetings, with Tukey’s honest significance test used to further evaluate the differences among groups. For comparisons between 2 groups such as between the use of video visits and in-person visits, the t-test was used. Two sided tests were used and *P*-values of <0.05 were considered to be significant.

We designed this study to be a retrospective cohort study and not a quality assessment or quality improvement project, thus review from the Rush University Institutional Review Board was sought and the study received exemption from full board review. Analysis were completed using RStudio, released September 2019.

## Results

**Figure 1.**
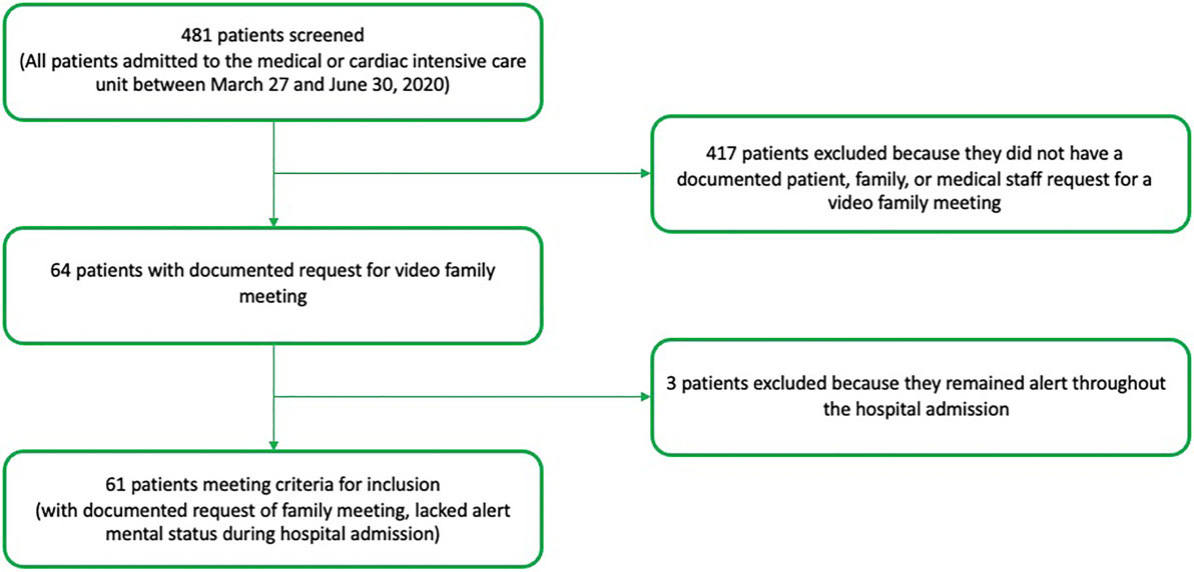
Inclusion criteria.

During the three-month study period between March 27 and June 30, 2020, 481 patients were admitted to the medical or cardiac intensive care unit. Patients were excluded if they did not have a documented request by patient, family, or medical staff for a video family meeting during their ICU stay (n =417) or remained alert throughout the entire hospital admission (n = 3). A total of 61 patients met inclusion criteria for this study (n = 61/481, 13%) (Figure 1).

### Participant Characteristics

Most patients were Hispanic or Latino (n = 32/61, 62%), followed by Black or African-American (n = 15/61, 25%) and White (n = 6/61, 10%) patients. The majority (n = 33/61, 54%) were English speaking, with a significant minority Spanish speaking (n = 26/61, 43%). Most patients died during hospital admission (n = 37/61, 61%), a quarter were discharged to a long-term acute care hospital (n = 15/61, 25%), and a few were discharged to hospice (n = 7/61, 11%). The primary reason for admission was COVID-19 pneumonia (n = 57/61, 93%). The mean length of stay was 23.5 days (SD 14.0), which did not vary significantly by race or ethnicity. Hispanic or Latino patients had a mean length of admission of 25.0 days (SD 13.9), Black or African-American patients of 19.9 days (SD 13.6), and White patients of 17.8 days (SD 15.2) (p = 0.23). Few patients had a documented health care power of attorney (n = 4, 7%), the remainder required health care surrogates assigned by state law (Table 1).

**Table 1.** Patient Characteristics (*N* = 61)^a^.

	Study Population
Age	
30-39	6 (10%)
40-49	5 (8%)
50-59	16 (26%)
60-69	18 (30%)
70-79	12 (20%)
80-89	4 (7%)
Race/Ethnicity	
Hispanic/Latino	38 (62%)
Black/African-American	15 (25%)
White	5 (10%)
Other	2 (3%)
Marital Status	
Married	31 (51%)
Single	21 (34%)
Widowed	5 (8%)
Divorced/Legally Separated	4 (7%)
Primary Language	
English	33 (54%)
Spanish	26 (43%)
Bilingual English/Spanish	1 (2%)
Insurance Status	
Medicaid	9 (15%)
Medicare	21 (34%)
Private	21 (34%)
Uninsured	10 (16%)
Admit Location	
Emergency Department	38 (62%)
Transfer from Acute Care Hospital or Long Term Care Hospital	17 (28%)
Transfer from Outside Emergency Department	6 (9%)
Discharge	
Expired	37 (61%)
Long Term Acute Care Hospital	15 (25%)
Hospice (inpatient)	5 (8%)
Hospice (home)	2 (3%)
Home	2 (3%)
Short term hospital	1 (2%)
Primary Diagnosis	
COVID-19 Pneumonia	57 (93%)
Other	4 (7%)
Mean Length of Stay	23.5 (14.0)
Mean Length of Stay Not Alert	18.1 (10.9)
Mean Time from Admit Until Not Alert	3.0 (3.9)
Decision-Maker	
POA	4 (7%)
Spouse (surrogate)	32 (52%)
Children (surrogate)	17 (28%)
Parents (surrogate)	7 (11%)
Siblings (surrogate)	4 (7%)

^a^ Statistics shown are mean and standard deviation for length of stay and time to not alert and quantity and percentage for all the other variables.

### Quantity and Mode of Family Meetings

A total of 650 family meetings were documented for the 61 patients included in this study. A minority of these meetings (n = 233/650, 36%) included discussion of patient goals of care. Most family meetings with discussion of goals of care included the patient’s legal health care power of attorney or surrogate, the decision-maker who is legally authorized to make decisions on the patient’s behalf if the patient is unable to make their own decisions (n = 217/233, 93%). In evaluating all family meetings, most occurred by telephone (n = 381/650, 59%) rather than video (n = 53/650, 8%) or in-person (n = 70/650, 11%). For some family meetings, the mode of meeting was not known due to lack of documentation in the medical record (n = 146/650, 22%). In evaluating goals of care family meetings, most meetings occurred by telephone (n = 109/233, 47%), when compared to video (n = 10/233, 4%) or in-person (n = 41/233, 18%).

**Figure 2.**
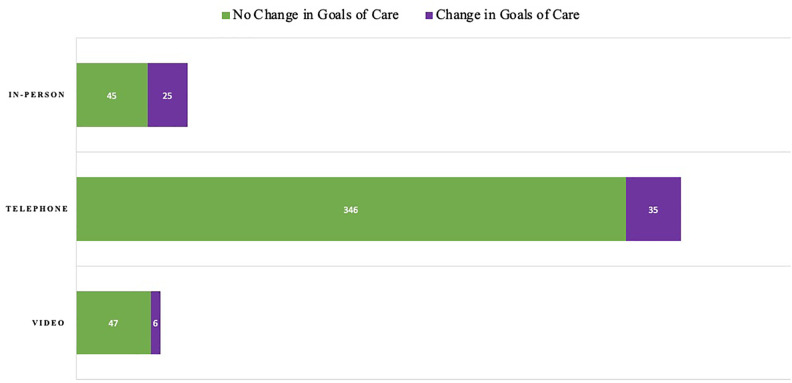
Change in Goals of Care in Family Meetings by Mode of Communication. This graph displays the quantity of family meetings discussing goals of care by mode of communication. It further differentiates these meetings into meetings where a change in the patient’s goals of care occurred, such as a decision to pursue hospice, versus a meeting where no change in goals of care occurred. When comparing modes of family meetings, a change in goals of care was more likely to occur for in-person meetings when compared to video meetings (p = 0.0006).

When evaluating the association of mode of family meetings by race, there was a higher mean quantity of documented telephone family meetings per patient admission for White (10.5, SD 9.5) and Black or African-American (7.1, SD 6.6) patients when compared to Hispanic or Latino patients (4.9, SD 2.9) (p = 0.02) (**Table 2**). The majority of in-person meetings included discussion of goals of care (n = 41/70, 57%). A quarter of telephone meetings included discussion of goals of care (n = 109/381, 29%), while a smaller percentage of video meetings included goals of care discussions (n = 10/53, 19%). When comparing modes of family meetings, a change in goals of care was more likely to occur for in-person meetings when compared to video meetings (p = 0.0006) **(** Figure 2).

**Table 2.** Mode of Family Meetings.

	All Patients, *n* = 61	Black/African-American, *n* = 15	Hispanic or Latino, *n* = 38	White, *n* = 6	Other, *n* = 2	*P*-value
Family Meetings per Patient Admission, mean (SD)						
Telephone Visits	6.2 (5.2)	7.1 (6.6)	4.9 (2.9)	10.5 (9.5)	12 (1.4)	**0.02**
Video Visits	0.9 (1.3)	0.8 (1.1)	0.8 (1.3)	1.7 (1.6)	0 (0)	0.36
In-Person Visits	1.5 (1.3)	1.1 (1.5)	1.1 (1.2)	1.7 (1.6)	1.5 (0.7)	0.75
Goals of Care Family Meetings per Patient Admission, mean (SD)						
Telephone Visits	1.8 (1.6)	1.5 (2.2)	1.2 (1.5)	1.6 (1.8)	0.7 (3.5)	0.21
Video Visits	0.2 (0.4)	0.3 (0.5)	0.1 (0.3)	0 (0)	0.5 (0.8)	0.08
In-Person Visits	0.7 (0.9)	0.6 (0.7)	0.7 (0.9)	0.8 (1.0)	0.5 (0.7)	0.94
Goals of Care Meetings with Change in Goals of Care per Patient Admission, mean (SD)						
Telephone Visits	0.6 (0.7)	0.4 (0.7))	0.6 (0.8)	0.8 (0.8)	0.0 (0.0)	0.40
Video Visits	0.1 (0.3)	0.2 (0.4)	0.0 (0.2)	0.3 (0.5)	0.0 (0.0)	0.05
In-Person Visits	0.4 (0.6)	0.4 (0.6)	0.4 (0.6)	0.7 (0.8)	0.0 (0.0)	0.59

SD = standard deviation.

*P*-value <0.05 are in bold.

### Timing of Family Meetings

The mean documented family meetings per patient admission was 9.1 meetings (SD 6.1) and mean number of goals of care family meetings was 3.0 meetings (SD 1.9) (Supplemental Table 1). On average, patients had a documented family meeting once every 2 days during their admission.

**Figure 3.**
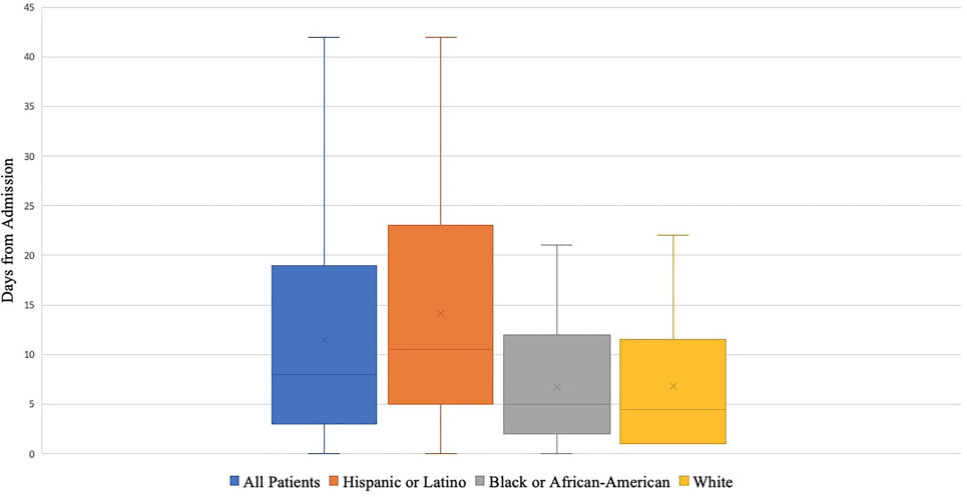
Time to First Documented Goals of Care Family Meeting. This graph displays the time to first goals of care family meeting for patients from hospital admission. It assesses time to first goals of care family meeting all patients, and also by race and ethnicity. A trend toward earlier mean first goals of care family meeting was noted for White (6.8 days, SD 7.9) and Black or African-American (6.7 days, SD 6.5) patients when compared to Hispanic or Latino patients (14.1 days, SD 10.8) (p = 0.06).

The mean time to the first goals of care family meeting was 11.4 days from admission (SD 10.1), and the mean time to first goals of care family meeting from loss of mental status of 8.4 days (SD 8.5). A trend toward earlier mean first goals of care family meeting was noted for White (6.8 days, SD 7.9) and Black or African-American (6.7 days, SD 6.5) patients when compared to Hispanic or Latino patients (14.1 days, SD 10.8) (p = 0.06) **(** Figure 3 **).**

### Participants in Family Meetings

The most frequent staff included in documented family meetings were critical care clinicians (n = 252/650 meetings, 39%). Bedside nurses were not often included in goals of care family meetings (n = 20/233 meetings, 9%), but were more frequently included in family meetings without goals of care discussion (n = 88/314 meetings, 28%) (Supplemental Figure 1).

### Palliative Medicine Involvement

Palliative medicine was consulted for 22 (36%) patients, with a mean time to consult of 18 days from hospital admission. Palliative medicine was included in a total of 43 family meetings for the patient population (n = 43/650, 7%). Patients with palliative medicine consults had a higher mean number of documented goals of care family meetings during admission than patients without a palliative consult (4.1 vs. 2.3 meetings, p = 0.0003).

## Discussion

During this period of strict visitor restrictions, family meetings continued to occur for patients in the intensive care unit with a minority of these meetings including discussion of patient goals of care. Few family meetings occurred in-person or by video and most occurred by telephone. When changes in goals of care occurred, the family meetings were more likely to have occurred in-person when compared to by video. White and Black or African-American patients had statistically significantly higher quantities of telephone family meetings than Hispanic or Latino patients. Documented goals of care family meetings occurred late into hospital admission with an average time to first meeting of 11.4 days from admission. Palliative medicine was not consulted on the majority of patients, and when consults occurred they often were placed late into a patient’s admission. More documented goals of care family meetings occurred when palliative medicine was consulted.

Our study found family meetings continued to occur during the COVID-19 pandemic with the majority occurring by telephone rather than in-person or by video. The quantity of family meetings during this study raise concern that the frequency of medical staff and family contact may be less than prior to the pandemic. Our study found the average number of family meetings per day patient was admitted was 0.5, which is less than the rate of 1.2 family meetings per day for ICU patients documented in a 2018 study.^12^ Although it is possible the rate of family meetings from this 2018 study are different than that occurring in the hospital where this study was performed prior to the pandemic, that concern is diminished as this study evaluated 14 different hospitals and thus more likely is generalizable. These findings are concerning because decreased family contact and family meetings may contribute to poor communication and may potentially affect patient mortality.^4,5^

We found documented goals of care family meetings by video led to fewer changes in goals of care for patients than in-person meetings. One reason for this finding may be that in-person meetings allow families to better understand the patient’s condition than video meetings, contributing to a higher likelihood that a change in goals of care occurs for the patient. It is also possible in-person meetings were more often used when the patient’s condition was declining when compared to video meetings, contributing to the higher changes in goals of care seen with in-person meetings.

Many patient families utilized video meetings during patient admission, however compared to telephone visits this method was not as frequently used as noted above. Video meetings can be a useful tool for discussion with patient families. One study evaluating the use of video family meetings by a tablet during the COVID-19 pandemic found families gave the meetings high ratings and felt comfortable asking questions and sharing their thoughts with the medical team.^13^ Guidance exists for clinicians regarding the implementation and use of video meetings to speak with patients and families; however, it is limited due to small sample size and lack of previous widespread use of video visits.^8,9^ There are barriers to video family meetings, as some populations do not have access or cannot use video devices such as the elderly or people who cannot afford them.^7,11^ This should be less of a concern for patients included in this study, however, as all patient families had access to extra assistance through case management to organize and explain how to use the video meetings.

This study found first documented goals of care family meetings occurred late, with the average time from admission to meeting of 11.4 days and average time from loss of mental status after admission to meeting of 8.4 days. Late documented goals of care meetings may be related to the uncertainty of treating patients with COVID-19 pneumonia and the length of time needed for some patients to recover.^14^ Increased patient caseload in the ICU during the study period may have decreased the likelihood that family meetings were documented as time needed to be spent on other patient care tasks.^15^ In addition to caring for patients admitted through the emergency department, the hospital where this study was conducted accepted hundreds of outside hospital transfers during this period adding to the increased workload for staff.^16^ Decreased documentation of meetings may be less likely though, as earlier family meetings were documented for certain patient populations such as White and Black or African-American patients. It is possible that the late timing of documented goals of care family meetings may not be related to COVID-19, as the timing of family meetings seen in this study is similar to a previous study conducted before the COVID-19 pandemic. This previous study evaluating patients in 2017 found 46% of intubated patients lacking decision-making capacity in a medical ICU had a family meeting within 3 days of admission, similar to the 41% of patients having a goals of care family meeting within 3 days seen in this study.^17^

Documented telephone encounters to update patient families or discuss goals of care did differ by race/ethnicity, with White patients having more documented calls per patient admission versus Black or African American and Hispanic or Latino patients. This is despite the fact that Hispanic or Latino patients had the longest length of admission on average compared to Black or African-American or White patients. All ICU teams were told they were required to provide daily updates to patient families for all patients in this study, so this difference in documented meeting rates based on race and ethnicity was unexpected. One possible reason for this difference is that Hispanic or Latino families often were Spanish speaking only requiring use of an interpreter. Spanish interpreters were easily accessible by telephone, but the extra step of including an interpreter to talk with families may have impacted the occurrence and documentation of these meetings. Some Hispanic or Latino families did not live in the United States and thus may have been more difficult to contact when compared to Black or African-American or White patients. However multiple documented notes show ICU teams were able to contact patient families in other countries. Any documented attempt at a meeting was included as an actual meeting in this study, making it less likely that difficulty contacting families contributed to the overall study findings. The variability in rates of contact with families by race and ethnicity are especially concerning as they may exacerbate already known COVID-19 disparities in Black and African-American and Hispanic or Latino populations.^18^

Excess US deaths during the COVID-19 pandemic resulted in some hospitals seeing increased need for the provision of palliative medicine for patients.^19,20^ Only a minority of patients in this study received a palliative consult and they occurred late in admission, which may have been associated with uncertainty of the course of COVID-19 in critically ill patients.^14,21^ A likely trigger for palliative medicine consults seen in this patient population, which on average occurred 18 days into admission, was consideration of placement of a tracheostomy. As guidelines including recommendations from the American Academy of Otolaryngology recommend waiting 2-3 weeks for placement of tracheostomies in patients with COVID-19, this timeline likely contributed to initiating the late consult to palliative medicine.^22,23^ Rate of palliative medicine consults potentially can be improved in the future with use of triggered palliative medicine consults which are associated with more transfers to hospice, decreased rates of tracheostomy placement, fewer days on mechanical ventilation, and no change in 30 day mortality.^24^

## Limitations

This study is limited in that only family meetings documented in the medical record were included. It is likely family meetings occurred for patients that were not documented, as all medical teams were informed they were required to complete daily updates to patient families. We believe most goals of care family meetings were documented in the medical record, as they were significant events that may have led to a change in care. This study population was at a single center, so the results may not be generalizable to intensive care units at other institutions. Another limitation is the limited sample size of this population. As this study was a retrospective cohort design, associations seen in this study may be impacted by confounding factors. Further research is needed to address this important point, especially evaluating racial and ethnic differences in use of family meetings. Severity of illness was not measured in this study limiting evaluation of illness impacting the use and mode of family meetings.

## Conclusions

During this period of strict visitor restrictions, few family meetings occurred in-person. Decreased rate of change in goals of care following video family meetings when compared to in-person family meetings appear to provide an association that restricting in-person family meetings may limit changes in patient care that would occur if families and alternate decision-makers were present. Low numbers of documented goals of care family meetings and differences in meeting rates based on race and ethnicity raise concern that there may be inadequate and unequal communication with families associated with visitor restriction policies. Further research in this area is needed to fully evaluate these concerns.

## Supplemental Material

Supplemental Material, sj-docx-1-ajh-10.1177_1049909120973431 - Family Meetings in the Intensive Care Unit During the Coronavirus Disease 2019 Pandemic Click here for additional data file. Supplemental Material, sj-docx-1-ajh-10.1177_1049909120973431 for Family Meetings in the Intensive Care Unit During the Coronavirus Disease 2019 Pandemic by Gina M. Piscitello, Corinna M. Fukushima, Anna K. Saulitis, Katherine T. Tian, Jennifer Hwang, Shreya Gupta and Mark Sheldon in American Journal of Hospice and Palliative Medicine®
