# Supplementary material for: Family Meetings in the Intensive Care Unit During the Coronavirus Disease 2019 Pandemic
Source: Am J Hosp Palliat Care. 2020 Nov 19;38(3):305–12. doi: 10.1177/1049909120973431 (PMC7859662; doi:10.1177/1049909120973431)
Supplement: Supplemental Material, sj-docx-1-ajh-10.1177_1049909120973431 - Family Meetings in the Intensive Care Unit During the Coronavirus Disease 2019 Pandemic [file sj-docx-1-ajh-10.1177_1049909120973431.docx]

**Electronic Supplemental Material**

**Manuscript Title:** Family Meetings in the Intensive Care Unit during the Coronavirus Disease 2019 Pandemic Appendix

**Author Names:** Gina M Piscitello MD, Corinna M Fukushima BA, Anna K Saulitis AM LCSW, Katherine T Tian MD, Jennifer Hwang BS, Shreya Gupta MPH, Mark Sheldon PhD

**Affiliation and email for corresponding author:** Rush Medical College, Gina_Piscitello@rush.edu

**Online Figure 1: Frequency of Attendance at Family Meetings**

I think this is already being done at Rush Oak Park, so we are just now starting to catch up about this.

**Online Table 1: Quantity, Rate, and Timing of Family Meetings**

|  | All Patients, *n*=61 | Black/African-American, *n*=15 | Hispanic or Latino, *n*=38 | White, *n*=6 | Other, *n*=2 | *P*-value |
| --- | --- | --- | --- | --- | --- | --- |
| **Quantity of Family Meetings, mean (SD)** |  |  |  |  |  |  |
| All Meetings | 9.1 (6.1) | 9.1 (7.0) | 8 (4.3) | 14.7 (11) | 13.5 (2.1) | 0.06 |
| Goals of Care Meetings | 3.0 (1.9) | 3.3 (2.3) | 2.7 (1.8) | 3.5 (2.3) | 4.0 (1.4) | 0.54 |
| **Rate of Family Meetings per Day (Length of Stay/Number Family Meetings), mean (SD)** |  |  |  |  |  |  |
| All Meetings | 0.5 (0.4) | 0.6 (0.6) | 0.4 (0.3) | 0.8 (0.2) | 0.4 (0.0) | **0.03** |
| Goals of Care Meetings | 0.2 (0.1) | 0.2 (0.1) | 0.1 (0.1) | 0.3 (0.1) | 0.1 (0.0) | 0.10 |
| **Rate of Family Meetings per Day Patient not Alert (Length of Stay Not Alert/Number Family Meetings), mean (SD)** |  |  |  |  |  |  |
| All Meetings | 0.6 (0.6) | 0.8 (0.6) | 0.6 (0.1) | 1.1 (0.3) | 0.4 (0.1) | 0.09 |
| Goals of Care Meetings | 0.2 (0.2) | 0.3 (0.2) | 0.2 (0.2) | 0.3 (0.2) | 0.1 (0.0) | 0.42 |
| **Timing of Family Meetings** |  |  |  |  |  |  |
| Time to First Family Meeting from Admission, mean (SD) | 2.7 (4.3) | 2.7 (2.8) | 2.6 (4.9) | 2.0 (2.3) | 6.5 (6.4) | 0.63 |
| Time to First Goals of Care Family Meeting from Admission, mean (SD) | 11.4 (10.1) | 6.7 (6.5) | 14.1 (10.8) | 6.8 (7.9) | 9.5 (10.6) | 0.06 |
| Time to First Goals of Care Family Meeting from Time Patient Not Alert, mean (SD) | 8.4 (8.5) | 4.5 (5.9) | 10.5 (9.1) | 5.2 (7.4) | 8.5 (10.6) | 0.10 |

SD= standard deviation

*P*-value <0.05 are in bold.
